# Plasma infliximab monitoring contributes to optimize Takayasu arteritis treatment: a case report

**DOI:** 10.1186/s40780-019-0136-4

**Published:** 2019-05-02

**Authors:** Sho Masui, Atsushi Yonezawa, Kazushi Izawa, Makoto Hayakari, Kayoko Asakura, Risa Taniguchi, Masahiko Isa, Hirofumi Shibata, Takahiro Yasumi, Ryuta Nishikomori, Junko Takita, Kazuo Matsubara

**Affiliations:** 1 Department of Clinical Pharmacology and Therapeutics, Kyoto University Hospital, 54 Shogoin Kawahara-cho, Sakyo-ku, Kyoto, 606-8507 Japan; 2 Graduate School of Pharmaceutical Sciences, Kyoto University, 54 Shogoin Kawahara-cho, Sakyo-ku, Kyoto, 606-8507 Japan; 3 Department of Pediatrics, Graduate School of Medicine, Kyoto University, 54 Shogoin Kawahara-cho, Sakyo-ku, Kyoto, 606-8507 Japan

**Keywords:** Infliximab, Pharmacokinetics, Takayasu arteritis, LC-MS/MS

## Abstract

**Background:**

Infliximab (IFX), a mouse-human chimeric monoclonal antibody against human tumor necrosis factor alpha, is used in refractory cases of Takayasu arteritis. Several factors influence the pharmacokinetics of therapeutic antibodies including IFX. Monitoring plasma levels of IFX could be a useful approach in optimizing treatment via individual dose adjustment.

**Case presentation:**

Here, we report the case of a 4-year-old Takayasu arteritis girl who was resistant to standard therapy. IFX was started at 5 mg/kg (day 0). C-reactive protein (CRP) levels decreased from 8.7 (day 0) to 1.6 mg/dL (day 10). CRP levels were thereafter elevated again on day 23 (9.0 mg/dL), and body fluid leakage at the inflammation site in the legs was observed. Trough IFX levels decreased from 23.6 (day 10) to 2.5 μg/mL (day 23). Based on the trough levels, IFX was given biweekly at 8 mg/kg. Plasma IFX levels gradually increased, and CRP levels decreased to around 2 mg/dL. A similar pattern -initial decreases followed by increases- was observed between clinical course of IFX and IgG levels. It was speculated that IgG and IFX losses were due to fluid leakage from the patient’s necrotizing legs.

**Conclusions:**

Monitoring of plasma IFX levels can be a potential tool to optimize the treatment in Takayasu arteritis patients.

## Background

Infliximab (IFX), a mouse-human chimeric monoclonal antibody against human tumor necrosis factor alpha (TNF-α), is used in the treatment of several autoimmune diseases. It has been reported that several factors influence the pharmacokinetics of therapeutic antibodies, such as development of anti-drug antibodies (ADAs) [1–3] and nephropathy [4]. Monitoring plasma IFX levels could be a potential tool for optimizing treatment via individual dose adjustment [5–7]. In fact, a tool (RemicheckQ®) with a similar purpose has been already approved for measuring blood concentrations of IFX. RemicheckQ® is a diagnostic kit used to determine whether serum IFX concentration is less or more than 1 μg/mL in patients with rheumatoid arthritis in Japan. However, monitoring of IFX levels is not common in other diseases. Takayasu arteritis is an autoimmune nonspecific large vasculitis affecting the aorta and its main branches with unknown etiology. Based on the Guidelines for Management of Vasculitis Syndrome [8] and reports [9–11], anti-TNF-α agents (such as IFX) are also used in refractory cases of Takayasu arteritis. Here, we report the case of a 4-year-old girl with Takayasu arteritis, in whom monitoring of plasma IFX levels was useful as a means of adjusting the regimen of IFX administration.

## Case presentation

A 4-year-old Japanese girl had fever and swelling in the right leg, with marked elevation of C-reactive protein (CRP) levels. Based on computed tomography, echocardiography and skin biopsy, she had been diagnosed with Takayasu arteritis at the age of two years. Due to aggravated inflammation, blood flow decreased in her legs, and part of her right leg became necrotic. As she had been resistant to standard therapy with prednisolone or tocilizumab without monitoring plasma concentrations, we started to administer IFX (day 0). IFX was given at a dose of 5 mg/kg on days 0 and 10. Although the levels initially decreased from 8.7 (day 0) to 1.6 mg/dL (day 10), CRP contents elevated again on day 23 (9.0 mg/dL), and IFX was administered at 10 mg/kg on the same day. Body fluid leakage from the inflammation sites in her legs was observed. Because blood IgG levels were lower than standard value, immunoglobulin (2.5 g) has been administered on days 17, 31, 37, 45, 51, 59, 65, 72, 85 and thereafter once a week for at least a few months.

**Fig. 1:**
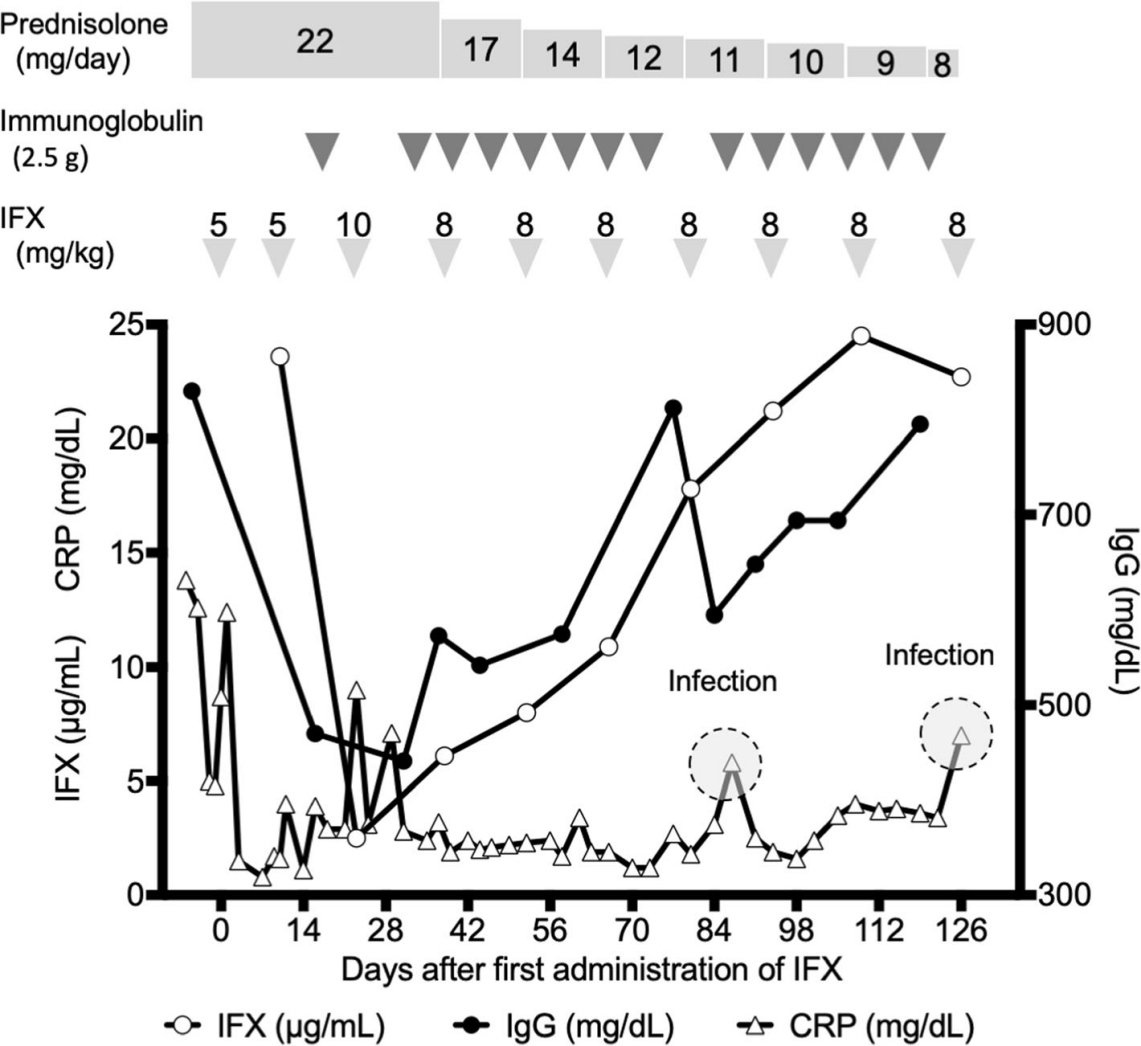
Clinical course of the patient. Trough plasma infliximab (IFX, open circle), immunoglobulin G (IgG, closed circle) and C-reactive protein levels (CRP, open triangle) levels are shown. IFX was administered at 5 mg/kg (days 0 and 10), 10 mg/kg (day 23), and thereafter at 8 mg/kg at 2-week intervals. Immunoglobulin (2.5 g) was administered on days 17, 31, 37, 45, 51, 59, 65, 72, 85 and thereafter once a week for at least a few months. Gray circles represent the escalation of CRP on days 87 and 126 due to infections

Plasma IFX concentrations were measured by LC-MS/MS with nano-surface and molecular-orientation limited (nSMOL, Shimadzu, Kyoto, Japan) proteolysis [12, 13]. Based on the clinical courses of blood CRP and IFX levels (Fig. 1), trough IFX levels were decreased from 23.6 μg/mL (day 10) to 2.5 μg/mL (day 23). Dosages and intervals of IFX administrations were then adjusted according to the trough IFX levels. IFX was given biweekly at 8 mg/kg per administration. Plasma IFX levels gradually increased, and CRP levels decreased to around 2 mg/dL 40 days after IFX administration. Inflammation was suppressed, and the dosage of prednisolone could be gradually decreased. CRP levels transiently elevated to 5.8 and 7.0 mg/dL after infection on days 87 and 126, respectively. Blood culture results confirmed the presence of gram-positive cocci. ADA against IFX was not detected using the enzyme-linked immunosorbent assay kit (Somru BioScience, PEI, Canada). During the observation period, no renal failure was observed. It is noteworthy to mention that a similar pattern -initial decreases followed by increases- was observed in the clinical courses of IFX and IgG (Fig. 1).

## Discussion

The present report described treatment of a young female Takayasu arteritis patient with IFX, resistant to standard therapy of tocilizumab without monitoring plasma concentrations. Because IFX therapy is an off-label use for the treatment of Takayasu arteritis, the treatment regimen was based on that for inflammatory bowel disease: i.e. 5 mg/kg at weeks 0, 2, 6 and subsequently at 8-week intervals. Wolbink et al. [14] have reported that the median (interquartile range) trough IFX levels in rheumatoid arthritis patients (dose: 3 mg/kg) at weeks 2, 6, 14 registered 22.3 (15.3–29.4), 14.6 (7.3–22) and 2.8 (0.6–6.8) μg/mL, respectively. However, in our case, the trough IFX levels were 2.5 μg/mL on day 23 (3–4 weeks) and 6.1 μg/mL on day 38 (5–6 weeks), even though IFX was administered at 5 mg/kg on days 0 and 10, and 10 mg/kg on day 23. It was suggested that IFX levels were too low to suppress the inflammation during this period. Finally, IFX administration at 8 mg/kg per 2 weeks succeeded in maintaining sufficient IFX levels to suppress the inflammation. This is the first report showing the relation to plasma IFX concentrations and effects on Takayasu arteritis. Monitoring of IFX levels was useful in the treatment of this patient with Takayasu arteritis.

Several factors affect pharmacokinetics of therapeutic antibodies, such as development of ADA and nephropathy. Many reports have indicated that consequent ADA formation due to an immunogenicity of IFX may decrease the functional drug concentration, resulting in a loss of response [1–3]. Counsilman et al. [4] have also reported that rituximab is rapidly excreted in the urine of a patient with severe nephrosis. However, ADA against IFX was not detected in the present patient, and no renal failure was also observed. Interestingly, there was an analogous tendency between the clinical courses of IFX and IgG levels. When both IFX and IgG levels decreased at around day 23, the patient had severe inflammation in her legs with substantial exudate. Although there were no previous reports, it was speculated that there was a loss of IgG (including IFX) due to the leakage from the necrotizing sites. Unfortunately, we could not collect the body fluid or measure IFX or IgG in it. Thereafter, a fluid leakage gradually decreased in association with wound healing, and the concentrations of both IFX and IgG elevated at the same dosage. This suggests that blood IgG level can be used as an index for monitoring IFX concentration.

## Conclusions

Inadequate IFX administration caused therapeutic failure. Monitoring of plasma IFX levels can be a useful approach in optimizing treatment of Takayasu arteritis patients.

## Abbreviations

ADA: anti-drug antibody
CRP: C-reactive protein
IFX: Infliximab
IgG: Immunoglobulin G
LC-MS/MS: Liquid chromatography tandem mass spectrometry
nSMOL: nano-surface and molecular-orientation limited
TNF-α: Tumor necrosis factor alpha
